# Is Chronic Kidney Disease a Critical Health Problem in Madeira Island?

**DOI:** 10.7759/cureus.46355

**Published:** 2023-10-02

**Authors:** Ana Carlota Vida, Nicole Pestana, Pedro Vieira, Gil Silva

**Affiliations:** 1 Nephrology Department, Hospital Central do Funchal, Funchal, PRT

**Keywords:** epidemiology and public health, prevention, portuguese population, prevalence, chronic kidney disease (ckd)

## Abstract

Chronic kidney disease (CKD) is an ever-growing global public health problem affecting more than 10% of the general population worldwide. CKD is associated with an increased risk of cardiovascular disease and all-cause mortality, representing a major burden to the healthcare system. Although multiple studies have determined CKD prevalence in different countries, there is still a significant knowledge gap between epidemiological surveys and real data recorded by healthcare providers. Regarding the Portuguese population, most recent studies revealed a CKD prevalence of 20.9%. However, there is an irregular distribution of CKD prevalence in the country. For example, considering the Madeiran population, a non-published review of lab results of nearly 130,000 patients in our database allowed us an estimation of 20%. Thus, to better comprehend CKD prevalence and its characterization in this region, we designed a study comprising previous studies’ strengths as well as aiming to overcome their limitations. The principal objective is to calculate global CKD prevalence in Madeira Island and stratify it by stage of CKD, allowing comparison of our results with recent literature on this subject. We intend to contribute with relevant epidemiological data in the characterization of CKD prevalence in Portugal and, simultaneously, have a more active role in CKD prevention and allocation of resources.

## Editorial

Chronic kidney disease (CKD) is an ever-growing global public health problem affecting more than 10% of the general population worldwide, corresponding to more than 800 million individuals [1]. CKD is associated with an increased risk of cardiovascular disease and an increased absolute risk of all-cause mortality, leading to a poorer quality of life and representing a major burden to the healthcare system. The annual number of deaths from kidney failure is 1.2 million, with an additional 1.4 million deaths due to cardiovascular disease attributed to CKD. Therefore, 4.6% of all deaths are due to CKD, making it the 12th leading cause of death [2]. In fact, surprisingly, when compared to non-metastatic cancer, mortality is similar for CKD during the first year of disease but 20% higher in the following years (one to five years) [3]. Older age, diabetes, hypertension, and obesity are among the most common risk factors. Concerning gender differences, CKD is more prevalent in women, but paradoxically, men receive more kidney replacement therapy due to kidney failure, which can be explained by the faster progression of CKD in men [1,4,5].

Multiple studies have been conducted to define CKD prevalence in different countries drawing awareness to this topic; however, there is still a significant knowledge gap between epidemiological surveys and real data recorded by healthcare providers. Filling this gap would help to adopt appropriate measures and interventions focused on the unmet needs of the CKD population. For this, an accurate assessment of CKD prevalence and progression could help downsize these negative impacts by allowing a proportionate dimensioning of healthcare resources for management and prevention. Nevertheless, this task may be harder than expected. Among different studies, the estimation of CKD prevalence is very distinct. When aiming for the rationale behind this irregular distribution, numerous reasons have been conducted. First, socioeconomic inequality and limited access to primary care and health services lead to some disparities between regions, which are even bigger when looking at whole countries. Second, a higher risk of developing CKD in some individuals as a result of comorbidities such as diabetes, arterial hypertension, cardiovascular or autoimmune diseases, older age, or the presence of family susceptibility to renal disease. All of these contribute to a population heterogeneity that may not be fairly represented in selected samples, thus limiting the obtained results.

European studies of CKD burden have shown marked variations in its prevalence across countries, varying from 3.3% to 17.3% [6]. Regarding the Portuguese population, in 2011, the PREVADIAB study from Vinhas’ workgroup revealed a 6.1% prevalence of CKD stages 3-5 [7]. Ten years later, the RENA study demonstrated a global CKD prevalence in Portugal of 20.9%, this time considering stages 1 to 5, with the highest prevalence found in the central region of Portugal (25%) and the lowest (12%) in Madeira Island [8]. More recently, a study in the north region of Portugal estimated a CKD prevalence (stages 1 to 5) of 9.8% [9]. In fact, there is an irregular distribution of CKD prevalence in Portugal, but there are also significant differences across these studies that might make comparisons difficult, as mentioned in Table 1. For example, the PREVADIAB study did not allow the establishment of chronicity when evaluating CKD, given that the evaluation of kidney function was not reassessed after three months [7].

**Table 1:** Comparison between different chronic kidney disease prevalence studies in the Portuguese population. CKD: chronic kidney disease; CKD-EPI: Chronic Kidney Disease Epidemiology Collaboration; eGFR: estimated glomerular filtration rate; MDRD: modification of diet in renal disease; PCHU: primary care health unit

	PREVADIAB (2011) [7]	RENA (2020) [8]	CKD prevalence in the North of Portugal (2022) [9]
Type of study	A cross-sectional study including 5,167 individuals with ages ranging between 20 and 79 years	A cross-sectional study including 3,135 individuals aged 18 or more and users of PCHUs. Social and demographic characteristics were collected by a self-report questionnaire. Chronicity was evaluated with measurement of eGFR at two different times	A cross-sectional study including 136,993 individuals aged 18 or more and users of 14 PCHUs assisted by Pedro Hispano Hospital. Chronicity was evaluated with measurement of eGFR at two different times
Equation used to calculate eGFR	MDRD equation	CKD-EPI equation	CKD-EPI equation and Cockcroft-Gault equation
Conclusions	CKD prevalence stages 3 to 5 of 6.1%	Global CKD prevalence stages 1 to 5 of 20.9%	Global CKD prevalence stages 1 to 5 of 9.8%
Limitations	Did not include albuminuria evaluation on protocol, not allowing for the prediction of CKD prevalence at stages 1 and 2. MDRD equation may have overestimated CKD stage 3. Excluded people older than 79 years which may have underestimated CKD global prevalence. Chronicity criteria, considering an eGFR ≤60 mL/minute/1.73 m^2^ for >3 months according to KDIGO guidelines, was not met	PCHU attendees typically present more comorbidities and are not representative of the Portuguese population. The studied population was predominantly Caucasian and with relatively old age. Absence of participation from the North and Algarve regions	The studied population was predominantly Caucasian. Population representative of northern Portugal, so the validity of results may not extend to the entire country

The Madeiran population is exceptionally suited for observational studies due to some particular characteristics, as stated before in our revision of native kidney biopsies [10]. This archipelago comprises about 250,000 inhabitants and has only one major medical facility making it a unique stage to assess the epidemiology and evolution of renal disease in this region. A retrospective non-published review of lab results of nearly 130,000 patients in our database allowed us to estimate a preliminary result of 20% CKD prevalence, considering a glomerular filtration rate (GFR) of less than 60 mL/minute/1.73 m^2^ estimated by the Chronic Kidney Disease Epidemiology Collaboration (CKD-EPI) formula based on Kidney Disease Improving Global Outcomes (KDIGO) guidelines [11]. Several explanations may contribute to this, such as increased susceptibility to develop kidney disease due to congenital or hereditary pathologies; socioeconomic factors, as people with lower socioeconomic status face a greater burden of CKD; the prevalence of comorbidities such as diabetes, arterial hypertension, dyslipidemia, and obesity which are known risk factors for CKD progression [1]; and the lack of awareness and education of the population to the high prevalence of kidney disease.

Considering all of this and to comprehend the true CKD prevalence and its characterization in this region, we designed a study comprising previous studies’ strengths as well as aiming to overcome their limitations. This cross-sectional, population-based study aims to include people aged 18 and older, stratified by age, sex, and district selected from a population register. Using the demographical data from Madeira Island, obtained in the 2021 Census, we aim to select a representative sample of each district by randomization. By doing this, we intend to screen the general population, overcoming the limitations stated in previous studies such as the RENA study, where only primary health users were evaluated [8]. People would then be asked by trained interviewers to participate in the study. Participants who would agree to take part would then be formally interviewed and clinically and analytically assessed at the local primary healthcare unit. Demographical data such as age, gender, ethnicity, profession, comorbidities, and chronic medications will be collected. Analytical evaluation would be based on blood and urine samples to determine serum creatinine, cystatin C, and albuminuria. For patients meeting CKD criteria, as classified in the KDIGO guidelines, a second evaluation would be assessed with a minimal three-month interval to confirm the first results and establish chronicity. By including measurements of cystatin C, we intend to reduce the CKD definition error, in consonance with more recent recommendations, where cystatin C evaluation is advised for initial diagnosis and staging, especially in groups under-represented in CKD-EPI collaboration cohorts, such as frail or fit [11]. The main objective of our study is to calculate global CKD prevalence in Madeira Island and stratify it by stage of CKD. Finally, we aim to compare our results with recent literature on this subject.

We have recently passed through a pandemic caused by SARS-CoV-2, with a global effect on our daily lives and a major impact on the way we see healthcare policies, particularly, when we deal with chronic patients, such as the CKD population. It was a challenging time, forcing us to reshape and adjust our strategies. On the other hand, the post-pandemic time brought us the energy and focus to move on to a new future and a world that places importance on preventive health strategies. In fact, by confirming the greater CKD prevalence previously referred, and considering the high cardiovascular risk and all comorbidities, to intervene earlier and delay renal disease progression. Preservation of kidney function can improve outcomes and can be achieved through non-pharmacological strategies (e.g., dietary and lifestyle adjustments) through patient awareness and chronic kidney disease-targeted and kidney disease-specific pharmacological interventions, which can be executed by timely referencing.

With this study we hope to contribute with relevant epidemiological data to the characterization of CKD prevalence in Portugal and, simultaneously, play a more active role in CKD prevention, helping to establish it as a health priority in the future.
